# Two new species of *Hymenaphorura* Bagnall, 1948 (Collembola, Onychiuridae) from Romania and an updated key to the genus

**DOI:** 10.3897/zookeys.960.50880

**Published:** 2020-08-17

**Authors:** Wanda Maria Weiner, Cristina Fiera

**Affiliations:** 1 Institute of Systematics and Evolution of Animals, Polish Academy of Sciences, Sławkowska 17, Pl–31-016 Kraków, Poland Institute of Systematics and Evolution of Animals, Polish Academy of Sciences Kraków Poland; 2 Institute of Biology Bucharest, Romanian Academy, 296 Splaiul Independenţei, P.O. Box 56-53, 060031 Bucharest, Romania Institute of Biology Bucharest, Romanian Academy Bucharest Romania

**Keywords:** Hymenaphorurini, identification key, morphology, taxonomy

## Abstract

Two new species of the genus *Hymenaphorura* from Romania, *H. urbana***sp. nov**. and *H. kalindera***sp. nov.**, are described and illustrated. *Hymenaphorura urbana* has a postantennal organ (PAO) with 13–15 simple vesicles, abdominal terga I–III with subequal setae p_2_ and p_3_, abdominal tergum V granular area with 3+3 distinct, long macrosetae, and *H. kalindera***sp. nov**. has PAO with 9–12 simple vesicles, one border seta, abdominal terga I–III with setae p_2_ slightly longer than setae p_3_, abdominal tergum V granular area with 4+4 distinct macrosetae. Remarks on *H. subsimilis* Bagnall, 1948 are given. An updated key for the world distributed species of *Hymenaphorura* is presented.

## Introduction

The genus *Hymenaphorura* Bagnall, 1948 is mainly characterized by two diagnostic apomorphies within the Hymenaphorurini: the presence of four guard setae on the antennal III sense organ and the lack of labial papillae E. Other characters of importance include: the absence of pseudocellus (pso) on the posterior part of the head, the body with only dorsomedial pseudocelli and the postantennal organ with simple vesicles, sometimes bilobed, located parallely or obliquely to the long axis of the organ, absence of the chaeta d0 on head, number of chaetae in the distal whorl of tibiotarsi (9 or 11), and structure of furcal rudiment (Pomorski 1998, 2001).

Of the 46 species of *Hymenaphorura* known globally (Bellinger et al. 1996–2020; Paśnik and Weiner 2018), five species have been recorded in Romania: *Hymenaphorura subsimilis* (Bagnall, 1948), *H. polonica* Pomorski, 1990, *H. nova* Pomorski, 1990, *H. valdegranulata* (Stach, 1954) (see Stan and Weisner 1978), and *H. ioni* Buşmachiu, Popa & Weiner, 2014.

During a study of some collembolan material collected in the last six years from Romania, two new species of *Hymenaphorura* were revealed and are described in this paper.

## Material and methods

### Sampling and preparation

Samples of leaf litter and soil were collected between 2013 and 2017 and extracted with Berlese funnels. The specimens were cleared in lactic acid and KOH and subsequently mounted on slides using Marc Andre II or Swan’s medium.

### Repositories

Collections are referred to the following acronyms:

**ISEA** Institute of Systematic and Evolution of Animals

**IBB** Institute of Biology Bucharest

**NHMUK** Natural History Museum UK

### Specimen examination

The taxonomic analysis was conducted using an Axio Scope A1 Zeiss microscope. Series of photographs were taken at different focal planes using an AxioCam ERC 5s camera mounted on microscope and processed with Adobe Photoshop CS3. Slide-mounted springtails were drawn using a Leica DM2500 compound microscope equipped with a camera lucida as well as phase-contrast and differential interference contrast (DIC) optical systems.

Labial papillae types are distinguished after Fjellberg (1999). Setae on the anal valves are named following Yoshii (1996). The nomenclature of the tibiotarsal chaetotaxy follows Deharveng (1983). Setae on furcal area are notated after Weiner (1996) and Paśnik and Weiner (2017). The pseudocelli, parapseudocelli, and pseudopores formulae give the number of pseudocelli, parapseudocelli, or pseudopores per half-tergum (dorsally) or half sternum (ventrally). The tibiotarsus chaetotaxy formula is expressed as the total number of setae (number of setae in row C, number of setae in row B, number of setae in row A+T), for example: 18 (1, 8, 9).

### Abbreviations

**Abd.** abdominal segments

**Ant.** antennal segments

**AS** anal spines

**AIIIO**Ant. III sensory organ

**bc** basal seta on maxillary palp

**^m^** unpaired pseudopore of Abd. II–IV sterna

**ms** microsensillum

**MVO** male ventral organ

**PAO** postantennal organ

**Th.** thoracic segments

**pso** pseudocellus

**psx** parapseudocellus

**psp** pseudopore

**d0** unpaired seta on head

## Taxonomy

### Hymenaphorura kalindera sp. nov.

Taxon classificationAnimaliaPoduromorphaOnychiuridae

BD177DBD-12F7-513B-B272-934FABF56792

http://zoobank.org/BBFE4452-F97D-4314-B851-79F1F4208F62

**Figure 1.**
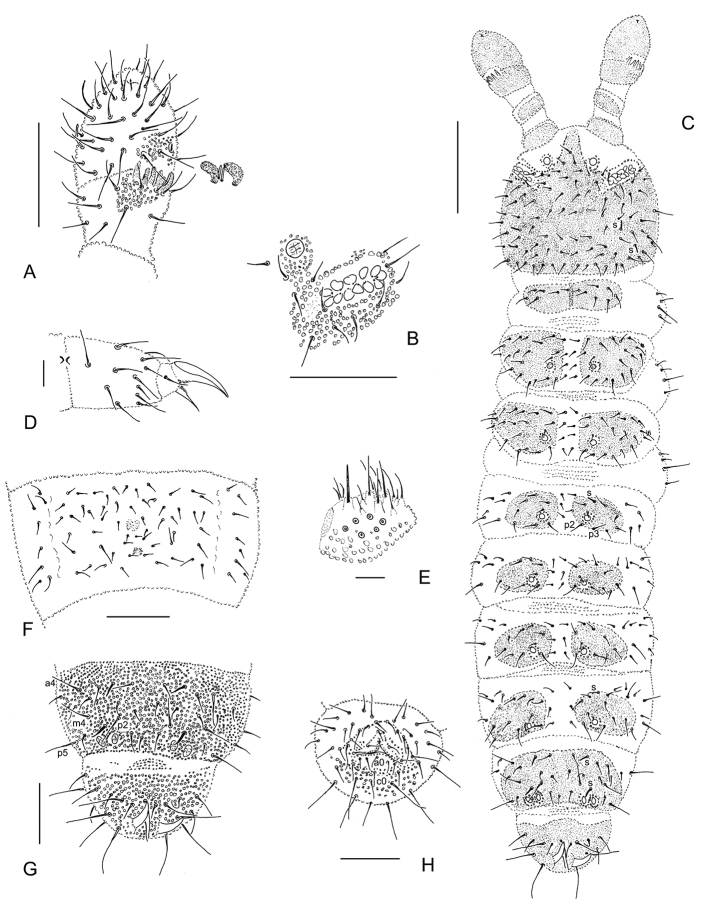
*Hymenaphorura kalindera* sp. nov. **A** antennal segment III and IV **B** postantennal sensory organ and anterior cephalic pseudocellus **C** habitus and dorsal chaetotaxy **D** leg III: tibiotarsal chaetotaxy and claw **E** labial palp **F** abdominal sternum IV with furcal rudiment **G** abdominal terga V and VI **H** abdominal sternum VI: anal valves. Scale bars: 0.05 mm (**A, B, F–H**); 0.01 mm (**B, E**); 0.1 mm (**C**).

**Figure 2.**
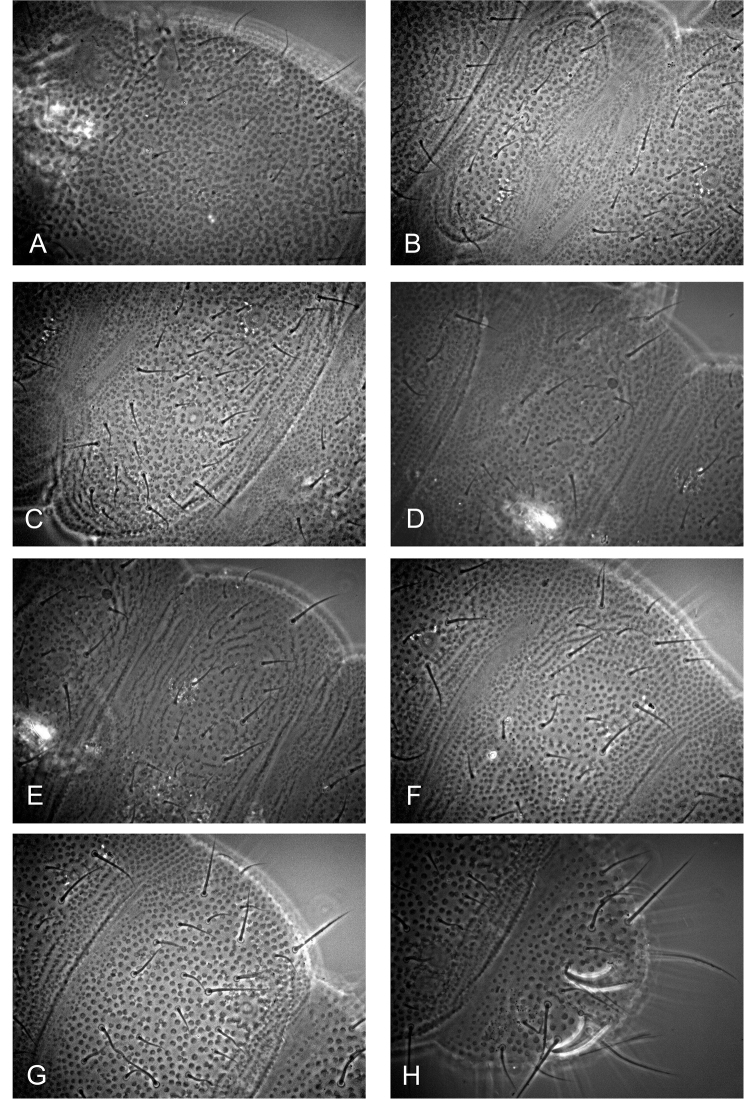
*Hymenaphorura kalindera* sp. nov.: dorsal chaetotaxy. **A** head **B** thoracic tergum I **C** thoracic tergum II **D** abdominal tergum II **E** abdominal tergum III **F** abdominal tergum IV **G** abdominal tergum V **H** abdominal tergum VI.

Figures 1A–H , 2A–H , Table 1

#### Material examined.

***Holotype***: female (IBB: RO-Hym1-IBB): Romania, Prahova County, Bucegi Massif, Buşteni near Kalinderu ski slope, 45.4212N, 25.52458E, 1000 m a.s.l., fir and beech forest, litter sample, 14.XI.2017, coll. C. Fiera. ***Paratypes***: female stored in Poland (ISEA: RO-17-1) and juvenile in Romania (IBB: RO-Hym2-IBB) same data as holotype.

#### Diagnosis.

Body with distinct areas of coarser granules. Dorsal pso formula as 10/011/11112, ventral pso absent. PAO with 9–12 simple vesicles, parallel or oblique in relation to the long axis of this organ and one border seta. Abd. terga I–III with setae p_2_ and p_3_ subequal. Abd. tergum V granular area with 4+4 distinct macrosetae. Distal tibiotarsal whorl with 11 setae.

#### Description.

***Measurements*** (in mm). Holotype female length 0.81, paratype female: 0.78, paratype juvenile 0.71.

***Body*.** Body elongate, cylindrical (Fig. 1C). Colour in alcohol white. Distinctive areas of granulation on dorsal side of the body of *c2* type (sensu Arbea and Jordana 1994). Usually 12–13 grains around each pseudocellus (Fig. 1B).

***Antennae and head.*** Antennae almost as long as head. Antennal segment I with 8 setae, antennal segment II with 15 setae. AIIIO consisting of four guard setae, five papillae, two smooth sensory rods, two granulated sense clubs: granulated and bent (Fig. 1A), ventro-lateral microsensillum present. Second external papilla in AIIIO forked in holotype, simple in two other specimens. Antennal segment IV with one distinct sensillum, small subapical organite in deep, narrow pit and latero-external microsensillum in the last posterior row of setae (Fig. 1A).

PAO with 8–12 beanlike vesicles, parallel or oblique in relation to the long axis of this organ in: holotype 10/12, paratypes 8/11 and 10/10 simple; PAO groove border with 1 seta (Fig. 1B). Labral formula of setae: 4/3,2,2. Maxillary palp simple with two sublobal sensory hairs. Labial type A (sensu Fjellberg 1999) with four papillae, papilla E absent (Fig. 1E). Guards a_1_, b_1–2_ and d_2_ (not well visible) as long as half of terminal sensilla of papillae. Five other guards as long as terminal sensilla.

***Pseudocellar formula.*** Pseudocellar formula per half tergum dorsally: 10/011/11112 (Fig. 1C), ventrally and on subcoxae 1 absent. Parapseudocelli and pseudopores not visible.

***Dorsal chaetotaxy.*** Dorsal chaetotaxy as in Figs 1C, 2A–H always with some asymmetry. Seta d0 on the head absent. Body with macro-and meso/microsetae, sensory setae s well distinguished on head, abdominal terga I, IV and V, their formula per half tergum: 2/000/10012.

Thoracic terga II and III with strong lateral microsensilla (ms). Thoracic tergum I with 7(6)+7(6) setae. Thoracic terga II and III with 5+5 macrosetae and 4+4 microsetae along midline. Abdominal terga I–III with 5+5 macrosetae and 3+3 microsetae along midline. Setae p_2_ and p_3_ on abdominal terga I‒III subequal. Granulated area of abdominal terga I–III with 4+4 setae, in row p of abdominal tergum V with 4+4 macrosetae as p_2_, p_5_, m_4_, a_4_. One macroseta in the set of setae on subcoxae 1 and abdominal pleura I–IV and 2 macrosetae on abdominal pleurum V. Abdominal tergum V with medial seta p_0_ (absent in juvenile), VI with medial setae a_0_ and p_0_. Anal spines as long as inner edge of claw III and 2.5 times as long as their basal diameter. Basal papillae low.

***Ventral chaetotaxy, furcal rudiment.*** Thoracic sterna II and III with 1 + 1 setae. Ventral tube with 7–9+7–9 setae. Male unknown. Abdominal pleurae II–V with 1, 2, 2, 2 macrosetae respectively. Abdominal sternum IV (Fig. 1F) with furcal rudiment as small finely granulated area and with two manubrial rows of setae: row ma with two setulae and row mp (irregular) with two macrosetae and 3–4 microsetae between them. Anal valves with numerous acuminate setae; each of lateral valves with three setae in a-row (a1-a0-a1) and five setae in b-row (b2-b1-b0-b1-b2), upper valve with one seta in a-row (a0), with four setae in b-row (b2-b1-b1-b2) and with three subequal setae in row c (c2-c0-c2) (Fig. 1H).

***Legs.*** Chaetotaxy of legs I, II and III as follows: subcoxae 1 with 4, 4, 4 setae, subcoxae 2 with 1, 4, 4 setae, coxae with 3, 9–10, 12 setae, trochanters with 8, 8, 9 setae, femora with 16, 14, and 14 setae, tibiotarsi with 3 whorls of setae: 20 (1,8,11), 20 (1,8,11), 19 (1,7,11) setae respectively (Fig. 1D). Seta M present. Claw without internal denticle. Empodial appendage with short basal lamella, length of empodium is about 2/3 of inner edge of claw III (Fig. 1D).

#### Ecology.

This species is found in litter samples in mixed fir and beech forest.

#### Etymology.

The name of the new species is inspired by the name of the ski slope: Kalinderu, Bușteni town, Prahova county, Romania.

#### Remarks.

*Hymenaphorura kalindera***sp. nov.** belongs to the group of species with 4+4 macrosetae on the granulated area of the abdominal tergum V (Table 1). *Hymenaphorura maoerensis* Sun, 2014, *H. rafalskii* Weiner & Szeptycki, 1997, and *H. wusuliensis* Sun & Wu, 2011 are different from this and other species by two border setae on the PAO. *Hymenaphorura rafalskii* and *wusuliensis* have 2+2 anterior pso on the head and 3+3 pso on the Abd. tergum V.

**Table 1.** Comparison of *Hymenaphorura* species with 4 macrochaetae on abdominal segment V.

**Species**	**PAO vesicles**	**Setae in tibiotarsal distal whorl**	**Setae on border of PAO groove**	**Labial type**	**Dorsal pso formula**	**Claw: inner denticle and lateral teeth**	**Abd. I‒III: p2/p3**	**Number of setae p in granulated area of Abd. I–III**	**Body size (mm)**	**Number of s-setae on abd. tergum V**
***H. kalindera* sp. nov.**	10‒12	11	1	A	10/011/11112	absent	p2 and p3 subequal	4	0.81‒0.87	2+2
***H. anatolii* Pomorski, 2001**	14–16	11	1	A**	10/011/11112	absent	subequal or p2 sometimes longer than p3	2	females 1.65–1.8 males 1.2–1.55	1+1
***H. gamae* Arbea & Jordana, 1994**	11‒12	11	1	?	10/011/11112	lateral teeth present	p2 shorter than p3	0	1.6–11.9	?
***H. ioni* Buşmachiu, Popa, Weiner, 2014**	15 (13‒14)	11	1	0	10/011/11112	lateral teeth present	subequal	2	1.51–1.65	1+1
***H. maoerensis* Sun, 2014**	8‒11	11	2	A	10/011/11112	absent	subequal	2	females 1.50‒1.75 males 1.40‒1.65	?
***H. palaearctica* Pomorski, 2001**	11‒14	11	1	A**	10/011/11112	lateral teeth present	subequal or p2 slightly longer	2	females 1.65‒2 males 1.5‒1.7	1+1?
***H. rafalskii* Weiner & Szeptycki, 1997**	9‒12	11	2	?	20/011/11113	denticle present, lateral teeth absent	subequal	2	1.08‒1.31	2+2
***H. subsimilis* (Bagnall, 1948)**	12‒13	11	1	A	10/011/11112	absent	subequal	3–4, 2, 2	1.25	1+1
*H. wusuliensis* Sun & Wu, 2011*	11‒13	9	2	A	20/111/11113	absent	subequal	?	females 0.87–1.05 male 0.78	2+2

*Sun and Wu 2011: figs 1, 7; Sun pers. comm. **Babenko pers. comm.

The other species: *H. anatolii*Pomorski 2001 (Russia, northern Palearctic), *H. gamae* Arbea & Jordana, 1994 (Spain), *H. ioni* Busmachiu, Popa & Weiner, 2014 (Romania), *H. palaearctica* Pomorski, 2001 (Russia), and *H. subsimilis* Bagnall, 1948 (Romania) form a group of more similar species with 11 setae in the tibiotarsal distal whorl, with the pso formula 10/011/11112, and with one setae on the border of the PAO. The new species differs from these species by the number of s-setae on Abd. V (2+2 vs 1+1), by the presence of four setae on each of the granulated areas of Abd. I–III and by its smaller size (Table 1). A comparison with *H. subsimilis*, a species described from the same county (Prahova), is rather difficult because only one type specimen is not in good condition (see Remarks for *H. subsimilis*). These species differ in their size: *H. subsimilis* is larger than *H. kalindera* sp. nov. (1.01 mm vs 0.81 mm), and *H. subsimilis* has Th. I with 4+4 setae (juvenile specimens?) in one row vs 7+7 or 6+7 setae in two rows in the new species. Abd. V has 1+1 s-setae in *H. subsimilis* vs with 2+2 s-setae in the new species. The shape of vesicles in PAO are transversally lobed in *H. subsimilis* vs bean-like in shape in the new species. The upper anal valve setae in c-row are equal in the new species and, in *H. subsimilis*, seta c0 is longer than setae c2.

### Hymenaphorura urbana sp. nov.

Taxon classificationAnimaliaPoduromorphaOnychiuridae

018EE4D9-3A7E-5EE4-91F7-E265318D60BC

http://zoobank.org/35D62DBF-71A7-48E7-81D5-3F4EEB6EB1EA

**Figure 3.**
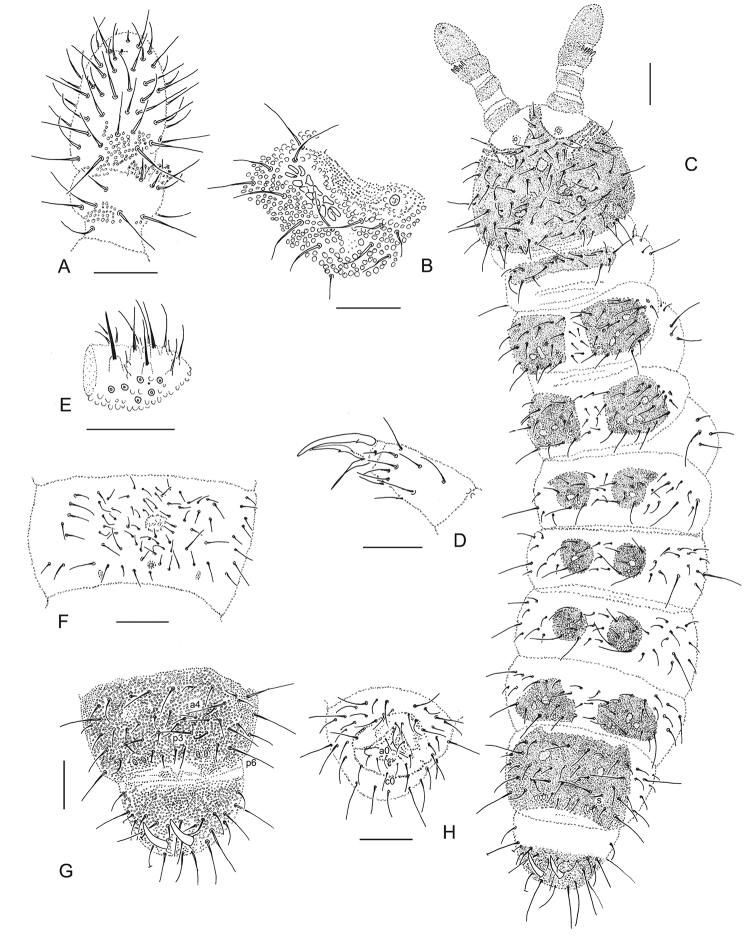
*Hymenaphorura urbana* sp. nov. **A** antennal segment III and IV **B** postantennal sensory organ and anterior cephalic pseudocellus **C** habitus and dorsal chaetotaxy **D** leg III: tibiotarsal chaetotaxy and claw **E** labial palp **F** abdominal sternum IV with furcal rudiment **G** abdominal terga V and VI **H** abdominal sternum VI: anal valves. Scale bars: 0.05 mm (**A, B, D–H**); 0.1 mm (**C**).

**Figure 4.**
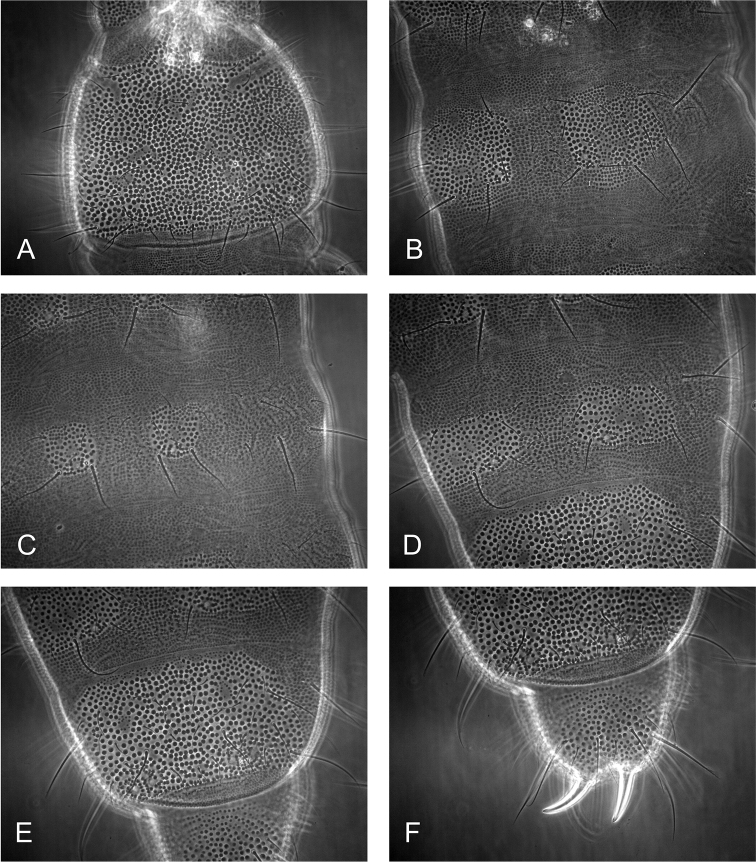
*Hymenaphorura urbana* sp. nov.: dorsal chaetotaxy. **A** head **B** thoracic tergum II **C** abdominal tergum II **D** abdominal tergum IV **E** abdominal tergum V **F** abdominal tergum VI.

Figures 3A–H , 4A–F , Table 2

Hymenaphorura nova – Fiera 2009: 871

#### Material examined.

***Holotype***: female (RO-Hym4-IBB): Romania, Bucharest, Cişmigiu park, soil under *Thuja orientalis* L., 44.4365N, 26.0901E, 72 m a.s.l., 05.XII.2013, coll. C. Fiera. ***Paratypes***: 3 females (2 in IBB: RO-Hym5,6-IBB and one in ISEA: RO-13-1), 2 males preadults (one in IBB: RO-Hym7-IBB and one in ISEA: RO-13-2), 2 juveniles (one in IBB: RO-Hym8-IBB and one in ISEA:RO-13-3), Bucharest, Cişmigiu park, same data as holotype.

#### Other material.

one male preadult and one female (IBB: RO-Hym9,10-IBB), Bucharest, Unirea park, 44,427980N, 26,101367E, 72 m a.s.l., 05.XII.2013, coll. C. Fiera.

#### Diagnosis.

Body with distinct areas of coarser granules. Dorsal pso formula as 10/011/11112, ventral pso absent. PAO with 13–15 simple vesicles, parallel or oblique in relation to the long axis of this organ (Fig. 3B) and one border seta. Abd. terga I–III with subequal setae p2 and p3. Abd. tergum V granular area with 3+3 distinct, long macrosetae. Distal tibiotarsal whorl with 11 setae.

#### Description.

***Measurements*** (in mm). Holotype female length 1.82, length of paratypes males: preadult 1.34–1.47, paratypes females: 1.50–1.88, females juvenile: 1.26–1.34.

***Body*.** Body elongate, cylindrical (Fig. 3C). Colour in alcohol white. Distinctive areas of granulation on dorsal side of the body of c2 type (sensu Arbea and Jordana 1994). Usually 9–11 grains around each pseudocellus (Fig. 3B).

***Antennae and head*.** Antennae almost as long as head. Antennal segment I with 8 setae, antennal segment II with 16 setae. AIIIO consisting of four guard setae, five papillae, two smooth sensory rods, two granulated sense clubs: ribbed and bent, ventro-lateral microsensillum present. Second external papilla in AIIIO not forked. Antennal segment IV without distinct sensilla, small subapical organite in deep, narrow pit and latero-external microsensillum last posterior row of setae (Fig. 3A).

PAO with 13–15 simple vesicles, parallel or oblique in relation to the long axis of this organ, PAO groove border with one seta (Fig. 2B). Labral formula of setae: 4/3,2,2. Maxillary palp simple with two sublobal sensory hairs. Labial type A (sensu Fjellberg 1999) with four papillae, papilla E absent (Fig. 3E). Small guards a_1_, b_1–2_, d_2_. Five other guards as long as terminal sensilla of papillae.

***Pseudocellar*, *Parapseudocellar*, *Pseudopores Formulae*.**Pseudocellar formula per half tergum dorsally: 10/011/11112 (Fig. 3C), ventrally and on subcoxae 1 absent. Parapseudocelli (psx) not always visible, their formula per half segment: 01/111/1111 dorsally and 111111^m^ ventrally. Subcoxae 1. with 2 psx each, each femur with one psx. Pseudopores 11/1111 dorsally. Abdominal sterna II–IV with one medial pseudoporus each: abdominal sternum II with pseudoporus between two rows of setae, abdominal sternum III with pseudoporus in posterior row, abdominal sternum IV with pseudoporus placed behind margin of manubrial area (below row p).

***Dorsal chaetotaxy*.** Dorsal chaetotaxy, always with some asymmetry, as in Figs 3C, 4A–F with macro- and meso/microsetae of different length. Seta d0 on the head absent, Sensory setae s very slightly marked, well differentiated on abdominal tergum V. Thoracic terga II and III with lateral microsensilla (ms). Thoracic tergum I with 8–12+8–12 setae (holotype: 8+10). Thoracic terga II–III with 7+7 fairly strong and subequal short macrosetae. Abdominal terga I–IV with 3+3 macrosetae, abdominal terga I–III with subequal p2 and p3. Abdominal tergum V with three long macrosetae (a4, p3 and p6). One macroseta in the set of setae on subcoxae 1 and abdominal pleura I–IV and 2 macrosetae on abdominal pleurum V. Abdominal tergum VI with medial setae a0, m0 and p0. Anal spines as long as inner edge of claw and 3 times as long as their basal diameter. Basal papillae low.

***Ventral chaetotaxy*, *furcal rudiment*.** Thoracic sterna II and III with 1–2 +1–2 setae respectively. Ventral tube with 8–12+8–12 setae (in holotype as 8+10). MVO in preadult males absent (adult males unknown).

Furcal rudiment with sternum with three irregular rows poorly distinguished comparing with other part of sternum. Setulae only sometimes distinguished (Fig. 3F). Basal papillae small as a half of width of spines. Each of even anal valves with 3 setae in row a (a1-a0-a1) and five setae in b-row (b2-b1-b0-b1-b2); upper valve with one seta in a-row (a0), four setae in b-row (b2-b1-b1-b2) and with three subequal setae in row c (c2-c0-c2) (Fig. 3H).

***Legs*.** Chaetotaxy of legs I, II and III as follows: subcoxae 1 with 4, 5(6), 5(6) setae, subcoxae 2. with 1, 5(4), 5(4) setae, coxae with 4, 11(10), 11(14) setae, trochanters with 11 (10), 11(10), 10 setae, femora with 17 (16), 16, and 14(15) setae, tibiotarsi with 3 whorls of setae: 20 (1,8,11), 20 (1,8,11), 19 (1,7,11) setae respectively. Seta M present. Claw without internal denticle, with pair of lateral teeth. Empodial appendage with small, narrow basal lamella, length of empodium is about ⅔ of inner edge of claw (Fig. 3D).

#### Ecology.

This species lives in the urban habitats of Bucharest.

#### Etymology.

The species name refers to the urban area where it was sampled (Latin, urbanus).

#### Remarks.

*Hymenaphorura urbana* sp. nov. belongs to the group of *Hymenaphorura* species with one seta in the PAO groove border, p2 seta subequal to p3 on abdominal terga I‒III, and three macrosetae on the granulated area of abdominal tergum V. The new species shares these characters (Table 2) with *H. alticola* (Bagnall, 1935), *H. arantiana* Weiner & Stomp, 2001, *H. improvisa* Pomorski & Skarżyński, 2000, *H. nearctica* Pomorski, 2001, *H. nicol*ae Barra, 1998, *H. nova* Pomorski, 1990, *H. polonica* Pomorski, 1990, *H. similis* (Folsom, 1917), and *yoshii* Paśnik & Weiner, 2018 (Table 2).

**Table 2.** Comparison of *Hymenaphorura* species with and 3+3 macrosetae on Abdominal segment V.

Species	PAO vesicles	Setae in tibiotarsal distal whorl	Setae on border of PAO groove	Labial type	Dorsal pso formula	Abd. I‒III: p2/p3	Body size (mm)
***H. urbana* sp. nov.**	13‒15	11	1	A	10/011/11112	subequal	females 1.64–1.92 males preadult 1.39–1.57
***H. alticola* (Bagnall, 1935)**	11(9‒16)	11	1	?	20/111/11112	p3 longer than p2	1.6‒2.0
***H. arantiana* Weiner & Stomp, 2001**	11‒13	11	1	A	10/011/11112	subequal	females 0.89‒1.18 males 0.77‒1.0
***H. improvisa* Pomorski & Skarżyński, 2000**	10‒11	9	2	AC	20/111/11112	p2 longer than p3	1.4‒1.7
***H. nearctica* Pomorski, 2001**	14‒16	11	2	?	10/011/11112	Subequal or p2 longer	females 1.8‒2.3 male 1.6
***H. nicolae* Barra, 1998**	12‒14	11	1	A	10/011/11112	p2 shorter than p3	1,9
***H. nova* Pomorski, 1990**	9‒11	11	1	0	10/011/11112	subequal	1,5‒2,2
***H. polonica* Pomorski, 1990**	10	11	1	A	10/011/11112	subequal	1.6‒2.1
***H. similis* (Folsom, 1917)**	8‒10	9	1 (rarely 2)	AC	10/011/11112	p2 longer than p3	females 1.5‒1.7 males 1.4‒1.5,
***H. yoshii* Paśnik & Weiner, 2018**	12‒15	11	1	A	10/111/11112	subequal or p2 slightly longer	females 2.0‒2.3 male 2.1

*Hymenaphorura urbana* sp. nov. differs of *H. nearctica* and *H. yoshii* by the presence of one seta on border of PAO groove vs two setae; it differs of *H. yoshii* and *H. improvisa* by the pseudocelar formula (10/011/11112 vs 10/111/11112) , of *H. improvisa* and *H. similis* by the number of setae in the tibiotarsal distal whorl (11 in the new species vs 9 in *H. improvisa* and *similis*). The new species differs of *H. nova* by the labial type (A vs 0 in *nova*) as well as by the number of vesicles in PAO (13‒15 vs 9‒11). Setae p_2_ and p_3_ are subequal in the new species and p_3_ is longer than p_2_ in *H. alticola* and *H. nicolae*. *Hymenaphorura urbana* sp. nov. differs of *arantiana* by length of empodial appendage, which length is equal with the inner edge of claw III and size (1.39‒1.92 mm for *urbana* vs 0.77‒1.0 mm for *arantiana*). The new species is most similar to *polonica*, but *H. polonica* has only 10 vesicles in PAO and *urbana* has 13‒15, and the granulation of Abd. tergum V is very coarse and presents cauliflower-like areas in *H. polonica*.

### Hymenaphorura subsimilis

Taxon classificationAnimaliaPoduromorphaOnychiuridae

Bagnall, 1948

D5A09FD5-718A-5B9C-BCB1-B9B5A0930737

**Figure 5.**
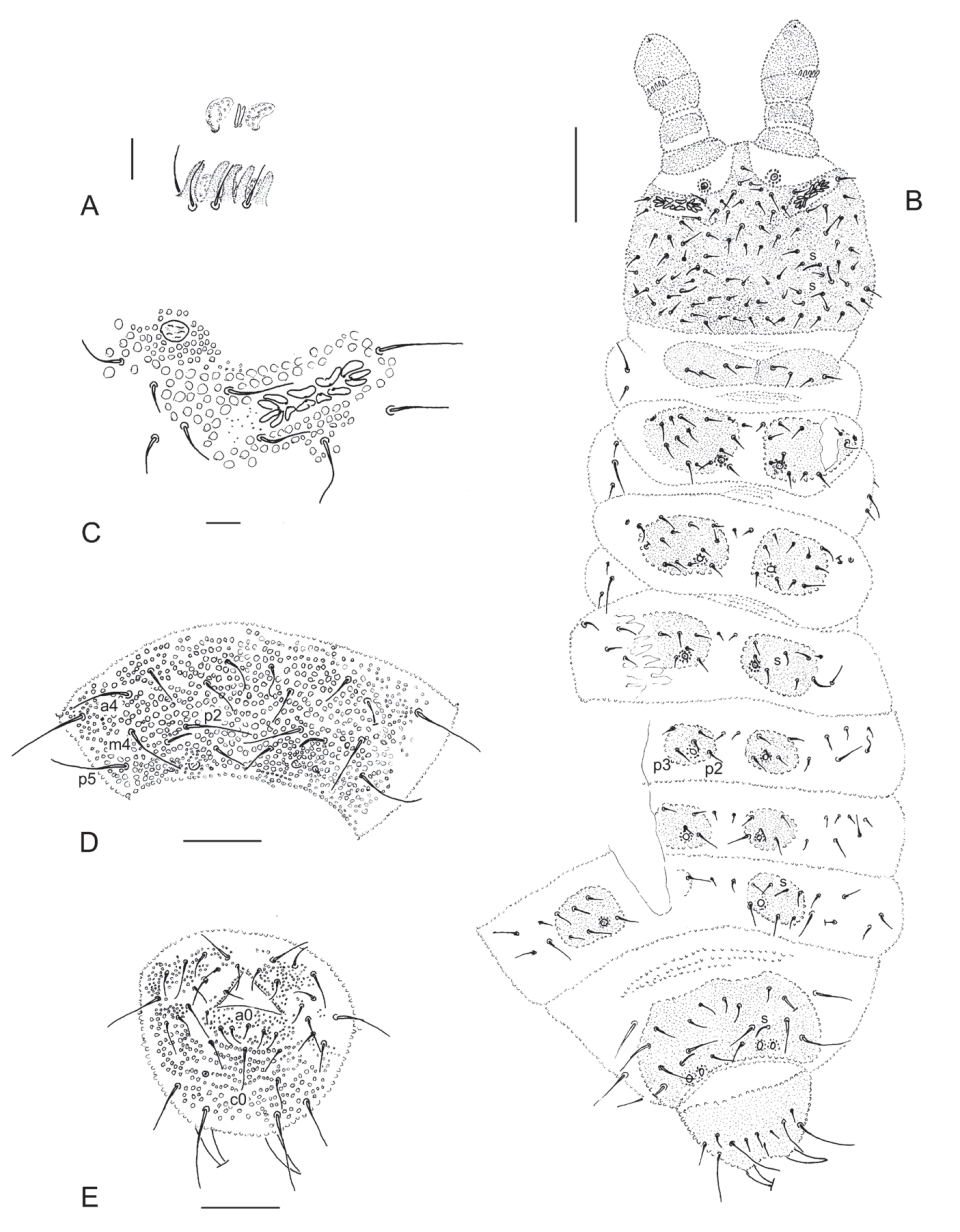
*Hymenaphorura subsimilis* Bagnall, 1948. **A** ant III sensory organ **B** habitus and dorsal chaetotaxy **C** postantennal sensory organ and anterior cephalic pseudocellus **D** abdominal terga V and VI **E** abdominal sternum VI: anal valves. Scale bars: 0.01 mm (**A, C**); 0.1 mm (**B**); 0.05 mm (**D, E**).

**Figure 6.**
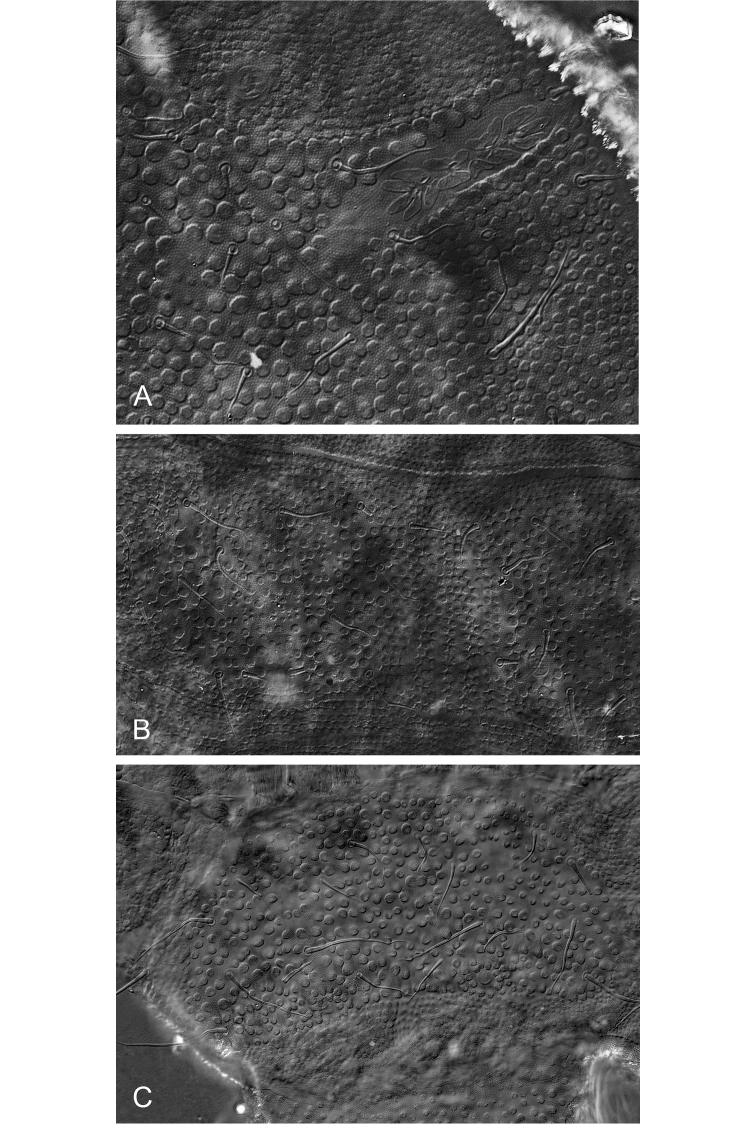
*Hymenaphorura subsimilis* Bagnall, 1948. **A** postantennal sensory organ and anterior cephalic pseudocellus **B** thoracic tergum II **C** abdominal tergum V.

Figures 5A–E , 6A–C , Table 1

#### Material examined.

Type specimen (NHMUK 012816837): Romania, Prahova, Sinaia, July 1934, coll. M. Manolache, among dead needles of *Larix*.

#### Complementary description.

***Antennae and head.***AIIIO consisting of four guard setae, five papillae, two smooth sensory rods, two granulated sense clubs: ribbed and bent (Fig. 4A). PAO with 12–13 simple vesicles (Fig. 5C, 6A). Labial type A (sensu Fjellberg 1999) with four papillae, papilla E absent.

***Dorsal chaetotaxy.*** Dorsal chaetotaxy as in Fig. 4B always with some asymmetry. Seta d_0_ on the head absent. Body with macro- and meso-microsetae and sensory setae s (slightly distinguished) on head, abdominal terga I, IV and V, their formula per half tergum: 2/000/10012.

Thoracic terga II and III with strong lateral microsensilla (ms). Thoracic tergum I with 4+4 setae. Thoracic terga II and III (Figs 5D, 6B) and abdominal terga I–IV with 1+1 microsetae (a1) along midline. Setae p2 and p3 on abdominal terga I‒III subequal. Abdominal tergum V (Figs 5D, 6C) with four long macrosetae (a4, m4, p2 and p5).

***Chaetotaxy of anal valves.*** Each of even anal valves with 3 setae in row a (a1-a0-a1) and five setae in b-row (b2-b1-b0-b1-b2); upper valve with one seta in a-row (a0), four setae in b-row (b2-b1-b0-b1-b2) and with three setae in row c (c2-c0-c2), c0 is distinctly longer than c2 (Fig. 5E).

Legs. Distal whorl of tibiotarsi with 11 setae. Empodial appendage with small, narrow basal lamella, length of empodium is about 2/3 of inner edge of claw.

#### Remarks.

In the Collembola collection in the Natural History Museum, London, only a single type microscope slide exists of *Hymenaphorura subsimilis*, which was described by Bagnall (1948). This slide is in a poor condition, with some characters not well visible or completely invisible. Salmon (1959) redescribed the type specimen, but without details of the chaetotaxy. We had an opportunity to study this type specimen, and we found that the description of *H. subsimilis* was probably based on a juvenile or anomalous specimen, because there is only seta a1 in the medial part of terga (Figs 6A, 5D) without m_1_ or p_1_ and also with only 4+4 setae on Th. I.

### Updated key to the known species of *Hymenaphorura*

The key presented below is based on the key by Paśnik and Weiner (2018). Recently described species have been added and some mistakes were corrected. *Hymenaphorura nova* has setae p_2_ and p_3_ on Abd. terga I–III subequal (not p_2_ shorter than p3 as according to Paśnik and Weiner 2018) and *H. anatolii* Pomorski, 2001 has setae p2 longer than p3 (not p2 is four times longer than p3 as described by Paśnik and Weiner 2018).

**Table:** 

1	Tibiotarsal distal whorl with 9 setae	**2**
–	Tibiotarsal distal whorl with 11 setae	**6**
2	Base of antenna with 1–2 pso	**3**
–	Base of antenna with 3 pso	***H. minuta* (Sun, 2014); China**
3	Base of antenna with 1 pso, PAO groove border with 1 or 2 setae	**4**
–	Base of antenna with 2 pso, PAO groove border with 2 setae	**5**
4	Th. I without pso	***H. reducta* Pomorski, 2001; North America**
–	Th. I with pso	***H. similis* (Folsom, 1917); USA, Siberia**
5	Granular area on Abd. tergum V with 3+3 macrosetae, Abd. terga I–III with p_2_ distinctly longer than p_3_, labial palpe of C type	***H. improvisa* Pomorski & Skarżyński, 2000; Poland**
–	Granular area on Abd. tergum V with 4+4 macrosetae, Abd. terga I–III with p2 and p3 roughly equal, labial palpe of A type	***H. wusuliensis* Sun & Wu, 2011; China**
6	Base of antenna with 1 pso	**7**
–	Base of antenna with 2 pso	**40**
7	Apex of Ant. IV with cauliflower-like papilla	**8**
–	Apex of Ant. IV without cauliflower-like papilla	**10**
8	Abd. terga I–III with setae p2 and p3 of roughly equal length or p2 slightly longer than p_3_	**9**
–	Abd. terga I–III with seta p2 about 4 times longer and thicker than p3	***H. ridibunda* Pomorski, 2001; USA**
9	Granular area on Abd. tergum V with 2+2 macrosetae	***H. cocklei* (Folsom, 1908); North America**
–	Granular area on Abd. tergum V with 4+4 macrosetae	***H. decus* (Christiansen & Bellinger, 1980); USA**
10	Th. I with 1 pso	**11**
–	Th. I without pso	**14**
11	PAO groove border with 1 seta, Abd. terga I–III with seta p_2_ and p_3_ of roughly equal length	**12**
–	PAO groove border with 2 setae, Abd. terga I–III with seta p_2_ about 4 times longer than p_3_	***H. alaskana* Pomorski, 2001; USA: Alaska**
12	Claw without inner denticle, granular area on Abd. tergum V with 1–3+1–3 macrosetae	**13**
–	Claw with inner denticle, granular area on Abd. tergum V with 6+6 macrosetae	***H. valdegranulata* (Stach, 1954); Europe**
13	Granular area on Abd. tergum V with 1+1 macroseta	***H. mystica* Pomorski, 2001; USA: Alaska**
–	Granular area on Abd. tergum V with 3+3 macrosetae	***H. yoshii* Paśnik & Weiner, 2018; Japan**
14	PAO with about 7–18 vesicles	**15**
–	PAO with 30–34 vesicles	***H. reisingeri* (Neuherz, 1979); Europe**
15	PAO groove border with 2 setae	**16**
–	PAO groove border with 1 seta	**20**
16	PAO with 16–18 vesicles	**17**
–	PAO with 8–11 vesicles	***H. maoerensis* Sun, 2014; China**
17	Seta p2 on Abd. terga I–III longer than p3.	**18**
–	Seta p_2_ on Abd. terga I–III equal or shorter than p_3_	**19**
18	Claw with denticle, seta p2 on Abd. terga I–III slightly longer, but thicker than p3	***H. nearctica* Pomorski, 2001; USA: Alaska, North-Eastern Asia**
–	Claw without denticle, seta p2 on Abd. terga I–III distinctly longer and thicker than p3	***H. sensitiva* Pomorski, 2001; USA: Alaska**
19	Seta p2 on Abd. terga I–III shorter than p_3_	***H. superba* Pomorski, 2001; USA: Alaska**
–	Setae p2 and p3 on Abd. terga I–III roughly equal	***H. granulata* Pomorski, 2001; USA: Alaska**
20	Seta p2 on Abd. terga I–III shorter than p3	**21**
–	Seta p2 on Abd. terga I–III equal, subequal or longer than p3	**23**
21	Granular area on Abd. tergum V with 4+4 macrosetae	**22**
‒	Granular area on Abd. tergum V with 3+3 macrosetae	***H. nicolae* Barra, 1998; Europe**
22	Claw with denticle, empodial appendage length equals to inner edge of claw, granulated area developed ‒‒type c1 according to Arbea & Jordana (1994)	***H. dentifera* (Stach, 1934); Europe (Carpathians and the Sudetes Mountains)**
–	Claw without denticle, empodial appendage length equals ⅔ of inner edge of claw, granulated areas on the body reduced ‒ type a according to Arbea & Jordana (1994)	***H. gamae* Arbea & Jordana, 1994; Europe: Spain**
23	Seta p2 on Abd. terga I–III distinctly longer than p_3_	**24**
–	Seta p2 and p3 on Abd. terga I–III equal or subequal	**25**
24	Two sublobal hairs on the maxillary outer lobe present	***H. anatolii* Pomorski, 2001; North Europe, Siberia**
‒	Sublobal hairs on the maxillary outer lobe absent	***H. inopinata* Babenko, 2017 in: Babenko et al. 2017, East European tundra**
25	Claw with inner denticle	**26**
–	Claw without denticle	**29**
26	Granular on Abd. tergum V area with 1‒3+1–3 macrosetae	**27**
‒	Granular on Abd. tergum V area with 6+6 macrosetae	**28**
27	Granular area on Abd. tergum V with 1(2)+1(2) macrosetae	***H. maiteae* Arbea & Jordana, 1994; Europe: Spain**
‒	Granular area on Abd. tergum V with 3+3 macrosetae	***H. nova* Pomorski, 1990 Europe**
28	Dorsal chaetotaxy with short setae and macrosetae poorly manifested	*H. sibirica* (Tullberg, 1877)^*^ ; **Siberia**
–	Dorsal chaetotaxy with long setae and very distinct macrosetae	***H. liberta* Pomorski, 1990; Crimea**
29	Granular area on Abd. tergum V with 1(2)+1(2) macrosetae	**30**
‒	Granular area on Abd. tergum with more macrosetae	**32**
30	Granulation of dorsal side of the body coarse, pseudocelli surrounded by 8–11 grains, size 1.3–2 mm	**31**
–	Granulation of dorsal side of the body with poorly visible granular areas, pseudocelli surrounded by 13–15 grains, empodial appendage length equals ¾ of inner edge of claw, small size 0.8–1.1 mm	***H. parva* (Skarżyński & Pomorski, 1996); Poland: Sudeten Mts.**
31	Empodial appendage length equals to inner edge of claw, pseudocelli surrounded by 9‒11 grains, 1.6–2 mm, males with MVO	***H. pseudosibirica* (Stach, 1954); Europe: Hungary**
‒	Empodial appendage length equals ½‒⅓ of inner edge of claw, pseudocelli surrounded by 8–9 grains, size 1.3–1.8 mm, males without MVO	***H. hispanica* Pomorski, 1992; Europe: Pyrenees**
32	Granular area on Abd. tergum V with 3+3 macrosetae	**33**
–	Granular area on Abd. tergum V with more macrosetae	**35**
33	Empodial appendage length equals ½–⅔ of inner edge of claw	**34**
‒	Empodial appendage length equals to inner edge of claw, small size (0.77–1.1 mm)	***H. arantiana* Weiner & Stomp, 2001; Europe: Luxembourg**
34	PAO with 10 vesicles, setae on the body rather short, granulation of Abd. tergum V very coarse with cauliflower-like areas	*H. polonica* Pomorski, 1990^*^; **Europe**
‒	PAO with 13–15 vesicles, setae on the body rather long, granulation of Abd. tergum V coarse, but without cauliflower-like areas	***H. urbana* sp. nov. Europe: Romania**
35	Granular area on Abd. tergum V with 4+4 macrosetae	**36**
–	Granular area on Abd. tergum V with 6–8+6–8 macrosetae	**39**
36	Granular area on Abd. tergum V with three lateral and one submedian macrosetae	**37**
–	Granular area on Abd. tergum V with two lateral and two submedian macrosetae	***H. palaearctica* Pomorski, 2001; Siberia**
37	Labial type A	**38**
–	Labial type 0	***H. ioni* Buşmachiu, Popa, & Weiner, 2014; Europe: Romania, Eastern Carpathians**
38	Abd. V tergum with one seta s (in row p) present, size 1.25 mm	***H. subsimilis* Bagnall, 1948; Europe: Romania**
‒	Abd. V tergum with two setae s (in row a and p) present, size 0.81‒0.87 mm	***H. kalindera* sp. nov. Europe: Romania**
39	Granular area on Abd. tergum V with 6(7)+6(7) macrosetae, granular area on Abd. tergum IV rather small with 10 setae (in row p only p_2_ and p_3_), empodial appendage length equals ⅔ of inner edge of claw	***H. alpina* (Stach, 1946); Europe: Alps**
–	Granular area on Abd. tergum V with 7(8)+7(8) macrosetae, granular area on Abd. tergum IV rather large with 13 setae (in row p 4–5 setae), empodial appendage length equals 4/5 of inner edge of claw	***H. teretis* Pomorski, 2001; USA: Alaska**
40	Th. I with pso	**41**
–	Th. I without pso	***H. rafalskii* Weiner & Szeptycki, 1997; North Korea**
41	Abd. terga I–III with p2 shorter than p3	***H. alticola* (Bagnall, 1935); Europe: Alps**
–	Abd. terga I–III with p2 and p3 roughly equal	***H. strasseri* (Stach, 1934); Europe: Slovenia**

## Conclusion

As we mentioned in the Introduction, 46 species and the two species newly described here belong to the genus *Hymenaphorura*. European species are the most numerous (28); there are 12 North American species and eight from the Far East. It is possible that such this pattern of species distribution is an artefact caused by the intensity of research in these regions. Further studies are needed to confirm.

Six species are insufficiently described: *H. californica* (Coleman, 1941), *H. jugoslavica* (Gisin, 1963), *H. montana* (Handschin, 1921), *H. submontana* (Denis, 1926), *H. troglodytes* Bagnall, 1948, and *H. uzicensis* B.P.M. Ćurčić, Lučić, S.B. Ćurčić & N.B. Ćurčić, 2005, and therefore they are not included in the key.

## Supplementary Material

XML Treatment for Hymenaphorura kalindera

XML Treatment for Hymenaphorura urbana

XML Treatment for Hymenaphorura subsimilis

## Footnotes

H. sibirica ‒ there is a discrepancy in the number of macrosetae on Abd. V given for this species. Pomorski (2001) has given 1+1 macroseta, while Weiner and Fjellberg (1994) in their redescription of H. sibirica have given 6 macrosetae on Abd. V.

H. polonica ‒ in the description Pomorski (1990) mentioned 1+1 poorly developed macroseta on Abd. V, while specimens from the type locality and other places have 3+3 macrosetae.
